# Lung Physiology and Obesity: Anesthetic Implications for Thoracic Procedures

**DOI:** 10.1155/2012/154208

**Published:** 2012-02-26

**Authors:** Alessia Pedoto

**Affiliations:** Department of Anesthesiology and Critical Care Medicine, Memorial Sloan-Kettering Cancer Center, 1275 York Avenue, Rm M301, New York, NY 10065, USA

## Abstract

Obesity is a worldwide health problem affecting 34% of the American population. As a result, more patients requiring anesthesia for thoracic surgery will be overweight or obese. Changes in static and dynamic respiratory mechanics, upper airway anatomy, as well as multiple preoperative comorbidities and altered drug metabolism, characterize obese patients and affect the anesthetic plan at multiple levels. During the preoperative evaluation, patients should be assessed to identify who is at risk for difficult ventilation and intubation, and postoperative complications. The analgesia plan should be executed starting in the preoperative area, to increase the success of extubation at the end of the case and prevent reintubation. Intraoperative ventilatory settings should be customized to the changes in respiratory mechanics for the specific patient and procedure, to minimize the risk of lung damage. Several non invasive ventilatory modalities are available to increase the success rate of extubation at the end of the case and to prevent reintubation. The goal of this review is to evaluate the physiological and anatomical changes associated with obesity and how they affect the multiple components of the anesthetic management for thoracic procedures.

## 1. Introduction

Obesity is a worldwide health problem. It is estimated that 34% of the North American adult population is obese, of which 5% is morbidly obese [1]. Thus, more patients requiring anesthesia for thoracic surgery will be overweight or obese. Anesthetic goals for thoracic procedures include a smooth induction and intubation, stable hemodynamic parameters during the intraoperative period, optimal lung isolation with adequate minute ventilation and good oxygenation,and optimal analgesia. However, being obese poses a challenge for all the above. The aim of this paper is to evaluate the physiological and anatomical changes associated with obesity and how they affect the anesthetic management for thoracic procedures. 

## 2. Physiological Changes and Comorbidities Associated with Obesity

### 2.1. Respiratory Mechanics

Obesity is associated with restrictive lung disease caused by increased intraabdominal pressure and decreased chest wall compliance [2, 3], resulting in a decrease in static and dynamic lung volumes [4] (Table 1). Low functional residual capacity (FRC) and expiratory reserve volume (ERV) contribute, respectively, to rapid desaturation with apnea or hypoventilation and air trapping with poor lung collapse during one-lung ventilation (OLV). This is more pronounced when ERV approaches or exceeds closing capacity. The decrease in FEV_1_ and FVC, which is inversely proportional to the increase in BMI [5], can affect postoperative respiratory function, especially after major lung resection or esophageal surgery. Finally, the decrease in lung and chest wall compliance may result in intraoperative hypoventilation and barotrauma during mechanical ventilation and increase the work of breathing in the postoperative period when patients resume spontaneous ventilation. These changes are worse in the supine position, under general anesthesia and during one-lung ventilation, and become more pronounced in the presence of a thoracotomy incision and lung parenchyma resection. This can cause further decrease in chest wall and lung compliance, as well as in all spirometric measures, starting from the immediate postoperative period and persisting for 6–8 weeks [6].

Spirometry has been used in the past to predict patients at risk for respiratory complications after major thoracic procedures, and it is still the mainstay to select candidates for major lung resection. The presence of poor baseline respiratory function, paired with extensive surgery, contributes to a high incidence of perioperative mortality, respiratory complications, and overall poor quality of life. In the past, FEV_1_ less than 1.5–2 L was considered a contraindication for lung resection (lobectomy and pneumonectomy), due to an increased mortality. However, more patients with marginal spirometry values are currently presenting for surgery. Major lung resection has been done in patients with an FEV_1_ less than 35% of predicted values with low postoperative mortality, especially if minimally invasive surgical techniques are used [7]. However, prolonged hospital stay and air leaks should be expected, especially when the FEV_1_ is less than 20%. Postoperative predicted FEV_1_ and diffusing lung capacity for carbon monoxide (DLCO) between 35 and 40% are considered the lower limit for resectability, especially if associated with a maximum oxygen consumption between 10 and 15 mL/kg/min. In this patient population, the use of regional analgesia techniques paired with short-acting intravenous anesthetics helps to increase the chance of extubation at the end of the case.

### 2.2. Obstructive Sleep Apnea

*OSA* is diagnosed in 5% of the obese population and is characterized by 10-second episodes of apnea (breathing cessation despite respiratory efforts against a closed glottis). The resulting chronic hypoxemia causes secondary polycythemia, hypercapnia, pulmonary, and systemic vasoconstriction and leads to an increased risk of cardiac and cerebral ischemia and intrapulmonary shunting [8, 9]. Patients with OSA show impaired pulmonary gas exchange both at rest and with exercise, and some studies have suggested an effect of male gender and adipose tissue distribution on the severity of this impairment [2]. Patients with OSA show larger decreases in lung volumes and compliance when anesthetized, and they are more prone to atelectasis and increased closing volumes and oxygen requirement [10]. This in turn contributes to rapid desaturation during induction of general anesthesia, especially in the absence of adequate preoxygenation or during difficult mask ventilation or intubation. A subgroup of patients with OSA can have *obesity hypoventilation syndrome (OHS)*. OHS is defined as chronic hypoventilation (PaCO_2_ > 45 mmHg) in the absence of lung, chest wall, and neurological disease [11]. Hypoventilation and apnea are central, and probably due to progressive desensitization to hypercapnia, which leads to an increased dependence on the hypoxic drive to maintain respiration. It affects 8–10% of patients with BMI > 30–34 kg/m^2^ and 18–25% of patients with BMI > 40 kg/m^2^ [11]. The presence of OSA or OHS should be screened in the preoperative evaluation, to optimize perioperative care. Several scoring systems have been developed for this purpose. The most commonly used system is the *STOP* (Snoring, day time Tiredness, Observed apnea, high blood Pressure)-*BANG* (BMI > 35, Age > 50; Neck circumference > 40 cm, Gender = male) questionnaire [12]. If patients answer yes to three or more questions, they are considered at high risk for OSA, and a sleep study should be obtained [12]. The use of continuous positive airway pressure (CPAP) at night can decrease apnea spells and reduce subsequent cardiac structural changes [13]. However, it is unclear how much time is necessary before a clinical improvement is seen. Patients using CPAP at home should continue to use it in the postoperative period as well, and arrangements should be made for continuous monitoring when discharged from the postoperative anesthesia care unit (PACU) to the floor. The presence of OSA should prompt customization of the anesthetic plan, by minimizing the use of long acting sedative medications, intravenous narcotics, and muscle relaxants. Regional techniques for postoperative analgesia should be favored if amenable.

### 2.3. Changes in the Upper Airway Anatomy

An increased amount of adipose tissue in the pharyngeal walls can result in upper airway collapse with spontaneous ventilation [9], causing difficulty in mask ventilation and/or intubation. Although the magnitude of BMI does not seem to correlate with difficult intubation, a thorough physical exam should be done to identify patients at risk. Signs suggestive of difficult mask ventilation or intubation include presence of a small mouth opening, short thyromental distance, increased neck circumference, decreased neck motility, and large breasts and tongue [4]. Furthermore, patient age, male gender, temporomandibular joint pathology, Mallampati 3 and 4, history of OSA, and abnormal upper teeth [14] are associated with difficulty in mask ventilation or intubation. Optimal positioning and advanced airway devices (such as fiberoptic bronchoscopes, glidescope, and laryngeal mask airways) should be available to facilitate securing the airway. Bronchial blockers and tube exchangers should also be readily available in case of difficult single-lumen tube placement.

### 2.4. Cardiac Changes

Obese patients are at increased risk for cardiovascular complications in the perioperative period [9]. Increased metabolic demand related to the excess of adipose tissue is responsible for the cardiovascular changes observed in obese subjects. Polycythemia and increased activity of the renin-angiotensin system cause an increase in total blood volume and cardiac output [15]. Hypervolemia paired with a high catecholamine tone can to lead to systemic and pulmonary hypertension, ischemic heart disease as well as left ventricular hypertrophy and dysfunction [4, 16], and an increased risk of atrial fibrillation and ventricular dysrhythmias [16]. The presence of hypoxemia and hypercapnia can further worsen pulmonary hypertension, resulting in right ventricular failure. Preoperative invasive cardiac testing may be necessary in these patients to assess their ability to tolerate lung resection and to guide the ventilator management during one-lung, ventilation. “Obesity cardiomyopathy” is characterized by systolic dysfunction and results in left ventricular dilatation. A summary of the main changes in cardiac physiology is listed in Table 2.

### 2.5. Other Comorbidities

Hypertension, hypercholesterole-mia, non-insulin-dependent diabetes mellitus, and poor exercise tolerance are all common in the obese patient. These patients are at increased risk of aspiration on induction because they commonly have gastroesophageal reflux, due to increased intraabdominal pressure, high acidic gastric volume, and decreased gastric motility [9]. Drug pharmacokinetics and pharmacodynamics are also altered in the obese population, affecting distribution and elimination of the drug. Dosing should be done by ideal body weight.

## 3. Obesity and Anesthetic Implications

Induction of general anesthesia causes a significant decrease in FRC, which is inversely related to the increased BMI [18]. A low FRC, paired with increased intrapulmonary shunt, decreased chest wall and lung compliance, increased airway resistance and atelectasis predispose the obese patient to rapid desaturation on induction [19, 20]. This may be aggravated by the abnormal upper airway anatomy, especially in the presence of OSA, causing difficult mask ventilation and intubation. The supine position causes a further decrease in FRC, due to both a cephalad displacement of the diaphragm and an increase in pulmonary blood volume. Both total respiratory and chest wall compliance are decreased when supine. Ventilation and perfusion are mismatched. As a result, relative hypoxemia is quite common and may persist in the postoperative period [21]. Patients should be placed in a semisitting position, preoxygenated for more than 5 minutes, and induced only if deemed easy to mask ventilate. If not, awake-fiberoptic intubation should be considered after topicalizing the airway with a local anesthetic. If sedation is needed, dexmedetomidine can be beneficial, as it has sedative and analgesic properties, with the lack of respiratory depression. Recruitment maneuvers, consisting of continuous positive pressure at 40 cmH_2_0 for 40 seconds, when used immediately after induction of general anesthesia seem to be successful at reducing the degree of atelectasis and improving oxygenation in normal weight and obese subjects [22], especially when followed by the immediate use of PEEP [10, 20].

### 3.1. Mechanical Ventilation and OLV

The choice of ventilation mode (pressure versus volume control ventilation) has become a very common dilemma both in the operating room and in PACU, due to an increased awareness and concern of ventilator-induced lung injury. Prolonged mechanical ventilation induces lung injury in critically ill patients by causing alveolar overdistention and increasing alveolar-capillary permeability (volutrauma), by releasing proinflammatory mediators (biotrauma), and by cyclic opening and closing of the alveoli (atelectrauma) [23]. As a result, respiratory morbidity and mortality are increased. The consensus from the ARDS-NET trial on mechanical ventilation has been to use low tidal volumes, with plateau pressures less than 30 cmH_2_O, the use of PEEP, and the acceptance of permissive hypercapnia [24, 25]. This has led to changes in clinical practice in the operating room as well. However, the use of low tidal volume and high FiO_2_ in the obese population remains controversial, since it can lead to progressive formation of atelectasis, with secondary hypoxemia and hypercapnia [26]. Conversely, high tidal volume could cause compression of the alveolar-capillary membrane, with subsequent worsening of gas exchange, especially during OLV [27]. Therefore the goal for mechanical ventilation in this patient population should focus on the use of peak inspiratory pressures high enough to open collapsed lung regions and the use of positive end-expiratory pressure to keep the alveoli open at the end of expiration [28]. The decrease in lung and chest wall compliance in obese patients may be protective against higher plateau pressures, with less lung damage during mechanical ventilation [20]. Ideal body weight should be used when setting the tidal volume on the ventilator, since lung size does not change with the increase in body weight [29].

When pressure (PCV) and volume control ventilation (VCV) were compared in obese patients (BMI > 35) undergoing laparoscopic gastric banding, the former was associated with an increase in pH, PaO_2_, and oxygen saturation, while PaCO_2_ was decreased [30]. It was postulated that because there is higher inspiratory flow with PCV, increased alveolar recruitment resulted in better ventilation/perfusion ratios. A positive effect on the hemodynamic parameters and a lower risk of barotrauma were also suggested with PCV. When these two modalities of ventilation were compared in healthy subjects with normal pulmonary function tests undergoing OLV, the only difference noted was lower peak inspiratory pressure (PIP) with PCV [31]. PIP is usually higher than peak alveolar pressure, since it depends on the resistance of the endotracheal tube and the breathing circuit. Therefore, it should not be used as a risk parameter to decrease barotrauma. A much stronger correlation exists with plateau airway pressure, especially if higher than 35 cmH_2_O. One disadvantage of PCV during OLV is hypoventilation, leading to further hypoxemia and hypercapnia, especially if there are changes in lung and chest wall compliance.

The intraoperative use of PEEP, with “optimal” values around 10–15 cmH_2_O, has been suggested to maintain the alveoli open [29], especially if used after a recruitment maneuver [20, 22]. In order to be successful, inflation pressure must be higher than alveolar opening pressure and be sustained [28]. However, this may not be tolerated in the presence of decreased preload, as seen in many thoracic surgical patients who are often hypovolemic or predisposed to cardiovascular disease [32].

### 3.2. Positioning

For thoracic surgical procedures, patients are commonly placed in the lateral decubitus position, which causes further changes in ventilation and perfusion. This position may be considered advantageous, especially in the presence of android obesity. The shifting of the abdominal pannus away from the diaphragm contributes to a decrease in intraabdominal pressure and greater chest excursions during mechanical ventilation [21]. Obese patients preferentially ventilate the nondependent lung when in the lateral decubitus both during spontaneous and mechanical ventilation [33]. Perfusion occurs mostly in the dependent areas of the lung and will decrease in the nondependent areas due to hypoxic vasoconstriction (HPV) and gravity-dependent changes. As long as appropriate minute ventilation is maintained, oxygenation during OLV in the lateral decubitus is usually satisfactory, especially when 100% FiO_2_ is used [21]. However, absorption atelectasis can develop over time and it should be considered when choosing the ventilator settings.

### 3.3. Extubation/Postoperative Management

Failed extubation and/or airway obstruction in the immediate postoperative period are common in obese patients, especially in the presence of COPD. The narrowing of the upper airway and the need to activate the pharyngeal muscles to achieve airway patency require patients to be awake at the end of the case. Furthermore, changes in compliance lead to an increased work of breathing (WOB) during spontaneous ventilation with subsequent rapid and shallow breathing pattern [5, 8]. Faster respiratory rates contribute to an increase in O_2_ consumption, dead space, and alveolar-arterial oxygen gradient [8, 17] which can be detrimental in the postoperative period. The use of noninvasive positive pressure techniques can increase the success of extubation or prevent reintubation in high-risk patients [4]. Specifically, continuous positive airway pressure (CPAP) and noninvasive ventilatory techniques (NIV) have been used successfully in obese patients. This can be helpful in case of major lung resection. However, there is some controversy about the use of CPAP and NIV after esophageal surgery, due to the concern of stomach insufflation and possible damage to the anastomosis. Judicious use of medications with sedative side effects, full reversal from neuromuscular blockade, and the use of regional analgesia can increase the success of extubation. Obese patients have poor respiratory muscle reserve which can contribute to an increased risk of respiratory failure under increased demand [29].

### 3.4. Analgesia

Optimal analgesia is important in the obese patient after lung resection. Rapid shallow breathing associated with poor analgesia, in combination with restrictive lung disease that characterizes obesity, is a risk factor for hypoxemia and possible respiratory failure. A multimodal analgesic approach is recommended, with particular emphasis on decreasing or avoiding medications that cause prolonged sedation. Regional analgesia has become more popular for thoracic surgery in the past years, in the form of thoracic epidural catheters (T5-8) or paravertebral blocks. Ideally, the placement should occur in the preoperative period. Intraoperative use of local anesthetic may increase the success of extubation in the operating room. However, epidural catheters and paravertebral blocks have the highest failure rate in the obese population [34]. Multiple attempts are often needed and may be associated with an increase in complication rates. Intraoperative use of adjuvant intravenous medications, such as *α*-2 agonists, nonsteroidal antiinflammatory agents, and acetaminophen, should be considered.

## 4. Postoperative Options for Assisted Ventilation

General anesthesia, surgery (especially high abdominal and thoracic procedures), as well as suboptimal analgesia, are all contributory factors to abnormal respiratory mechanics in the postoperative period, which may persist for several days [28]. The resulting restrictive respiratory pattern seen in obese patients contributes to an increased work of breathing and the tendency to develop atelectasis. Therefore, weaning from the ventilator may be prolonged, increasing the rate of ICU admissions and the overall hospital length of stay [35]. The rate of reintubation may also be higher when compared to normal weight patients. Noninvasive ventilation modalities have been developed trying to decrease the incidence of postoperative respiratory complications and avoid reintubation in the immediate postoperative period.

*Non invasive ventilation (NIV)* refers to techniques used to support respiration without the use of endotracheal intubation [36]. This can be either a preventive or a curative maneuver, once respiratory failure has occurred. The main goals of NIV are to (1) decrease the work of breathing, (2) increase alveolar recruitment, and (3) decrease left ventricular afterload with an increase in cardiac output and an improvement in hemodynamics [36]. Two modalities have been described to achieve these goals: CPAP (continuous positive end-expiratory pressure) and NPPV (noninvasive positive pressure ventilation).

1. *Continuous positive airway pressure (CPAP)* therapy is often used in obese patients after extubation in the recovery room to improve recruitment of atelectatic lungs and maintain patency of the upper airway [4]. In the immediate postoperative period, the risk of airway obstruction is high due to the anatomical changes associated with obesity and possible residual anesthesia, neuromuscular blockade, or suboptimal or excessive analgesia. This risk is increased in the presence of OSA or hypopnea syndrome [37]. The decrease in both VC and FRC contributes to an increase in atelectasis, which continues to worsen during the first 24 hours after surgery [38]. The use of CPAP during preoxygenation at the beginning of a general anesthetic and in the immediate postoperative period has been shown to improve respiratory mechanics and lung function during the hospital stay [37]. CPAP is done by using continuous positive pressure applied to the nose or the mouth by a machine. The settings chosen are usually titrated to upper airway patency [11] and can be empirically set to a start value corresponding to what is used at home. Unfortunately, this technique is underutilized in PACU and is better tolerated by patients who are already using a CPAP machine at home. Theoretical advantages of CPAP include promoting airway patency and preventing alveolar collapse, with a decrease in the FiO_2_ needed and a decrease in absorption atelectasis. Left ventricular afterload is also decreased, with an improvement in cardiac output [36].
2. *Noninvasive positive pressure ventilation (NPPV)* combines the use of pressure support ventilation (PSV) and positive-end expiratory pressures (PEEPs). Both methods have been used to prevent acute respiratory failure in the immediate postoperative period or to treat respiratory insufficiency, in attempt to avoid endotracheal intubation [39]. In the first hours after surgery, there is a 20% decrease in tidal volume, paired with a 20% increase in respiratory rates and diaphragmatic dysfunction. This can persist for one week [36]. The resulting alveolar hypoventilation paired with atelectasis can contribute to the development of pneumonia. The rationale for the use of NIV is to decrease the work of breathing by improving ventilation and to decrease the amount of atelectasis, with subsequent improvement of gas exchange [36]. By allowing independent adjustments of the inspiratory and expiratory pressures, NIV is used in case of hypoventilation syndromes to increase CO_2_ removal. The use of end-expiratory pressure promotes upper airway patency while the inspiratory component is useful to control central hypoventilation [4]. NIV techniques have been used both as a prophylactic treatment after lung resection and a curative method with no air leaks at the chest tube sites [36]. Moreover, oxygenation and lung volumes seem to improve during the first three postoperative days, when compared to the controls, with a decrease in length of stay in the hospital [36]. When used as a treatment option after lung transplantation, a decrease in the reintubation rates has been observed [36]. Patient cooperation is crucial in order for this technique to be successful. Starting with low PEEP and then adding PSV can be helpful. NIV is contraindicated in case of upper GI surgery, where it could contribute to leaks at the anastomosis site [36]. Failure of NIV has been demonstrated in patients after major lung resection when unable to clear secretions therefore requiring multiple bronchoscopies [40]. Increased secretions can cause an increased work of breathing and decrease the efficacy of NIV. Any delay in recognizing NIV failure and endotracheal intubation can lead to an increased morbidity and mortality, therefore a high vigilance and strict monitoring are necessary.

## 5. Conclusions

Obese patients have significant changes in static and dynamic respiratory mechanics, as well as multiple preoperative comorbidities, which should be considered when providing general anesthesia. The preoperative evaluation should be tailored to identify patients at risk for difficult ventilation and intubation and postoperative complications. The analgesia plan should be executed starting in the preoperative area. Intraoperative ventilatory settings should be customized to the changes in respiratory mechanics for the specific patient and procedure. Several non invasive ventilatory modalities are available to increase the success rate of extubation at the end of the case and to prevent reintubation. However, they are not free of risks and complications. A high index of suspicion for postoperative respiratory complications is necessary prior to the start of the case, as well as a thorough multidisciplinary approach to the perioperative care in order to optimize outcomes.

## Figures and Tables

**Table 1:** Changes in static and dynamic lung volumes in obese patients [4, 5, 8, 17].

Respiratory changes		
Decreased	Unchanged	Increased
FRC	FVC/FEV_1_	WOB
ERV		VO_2_
FEV_1_		DLCO
FVC		
C_L_		
C_CW_		
MMV		
VC		
TLC		
RV		

Abbreviations: FRC: functional residual capacity; ERV: expiratory reserve volume; FEV_1_: forced expiratory volume; FVC: forced vital capacity; C_L_: compliance lung; C_CW_: compliance chest wall; MMV: maximum voluntary ventilation; VC: vital capacity; TLC: total lung capacity; RV: residual volume; WOB: work of breathing; VO_2_: maximum oxygen consumption; DLCO: diffusing lung capacity for carbon monoxide.

**Table 2:** Cardiac changes associated with obesity [9].

Cardiovascular changes		
Decreased	Unchanged	Increased
	Cerebral blood flow	Blood volume
	Renal blood flow	Cardiac output (stroke volume)
		Oxygen consumption
		CO_2_ production
		Cardiac work

Abbreviations: CO_2_: carbon dioxide.
